# Heterotrophic Production of Omega-3 Long-Chain Polyunsaturated Fatty Acids by Trophically Converted Marine Diatom *Phaeodactylum tricornutum*

**DOI:** 10.3390/md14030053

**Published:** 2016-03-09

**Authors:** Mary L. Hamilton, Stephen Powers, Johnathan A. Napier, Olga Sayanova

**Affiliations:** mary.hamilton@rothamsted.ac.ukstephen.powers@rothamsted.ac.ukjohnathan.napier@rothamsted.ac.uk

**Keywords:** microalgae, *Phaeodactylum tricornutum*, omega-3 long chain polyunsaturated fatty acid, EPA, DHA, heterotrophic

## Abstract

We have created via metabolic engineering a heterotrophic strain of *Phaeodactylum tricornutum* that accumulates enhanced levels of the high value omega-3 long chain polyunsaturated fatty acid (LC-PUFAs) docosahexaenoic acid (DHA). This was achieved by generation of transgenic strains in which the Δ5-elongase from *Ostreococcus tauri* was co-expressed with a glucose transporter from the moss *Physcomitrella patens*. This double transformant has the capacity to grow in the dark in liquid medium supplemented with glucose and accumulate substantial levels of omega-3 LC-PUFAs. The effects of glucose concentrations on growth and LC-PUFA production of wild type and transformed strains cultivated in the light and dark were studied. The highest omega-3 LC-PUFAs accumulation was observed in cultures grown under mixotrophic conditions in the presence of 1% glucose (up to 32.2% of total fatty acids, TFA). Both DHA and EPA are detected at high levels in the neutral lipids of transgenic cells grown under phototrophic conditions, averaging 36.5% and 23.6% of TFA, respectively. This study demonstrates the potential for *P. tricornutum* to be developed as a viable commercial strain for both EPA and DHA production under mixo- and heterotrophic conditions.

## 1. Introduction

Microalgal strains have enormous potential to contribute to the bio-economy from production of low value products such as animal feed and biofuels to high value food supplements and nutraceuticals [1,2]. It has long been recognised that *n*-3 (omega-3) long chain polyunsaturated fatty acids (LC-PUFAs) have beneficial effects on human health. Eicosapentaenoic acid (EPA; 20:5*n*-3) and docosahexaenoic acid (DHA; 22:6*n*-3) are essential constituents of human nutrition with key roles in growth and development in infants and cardiovascular health in adults [3,4]. To satisfy the demand for these nutrients, cost effective production systems have to be developed. Marine microalgae represent a promising recourse for biotechnology of LC-PUFA production. Many photosynthetic species of microalgae, such as *Nannochloropsis*, *Monodus subterraneus*, *Phaeodactylum tricornutum*, *Odontella aurita* and *Porphyridium cruentum* have the ability to synthesize high levels of EPA and lower levels of DHA. DHA-producers include mainly heterotrophic microalgae such as the thraustochytrids *Aurantochytrium* spp. [5], *Thraustochytrium* spp. [6,7], *Schizochytrium* spp. [8] and the dinoflagellate *Cryptocodinium cohnii* [9]. However, only few algal species that accumulate have the ability to produce high levels of both EPA and DHA. At present, mostly thraustochytrids are used in industrial-scale *n*-3 LC-PUFA production via heterotrophic cultivation [10].

In spite of intensive research over the last few decades, a number of bottlenecks exist which must be overcome for algae to be a viable source of these products. Key hurdles to the commercialisation of products from microalgae are the high cost of producing biomass and the efficient extraction of valuable products [11].

Most microalgae are obligate photoautotrophs and require light for their growth. At present, the most common procedure for cultivation of microalgae is autotrophic growth in open ponds and closed photobioreactors (PBR). The requirement for light to increase cell densities and production is the main objective and a limiting factor of the cultivation. Photoautotrophic growth of algae requires low inputs but cellular growth is limited by light availability which restricts biomass. Light penetration is inversely proportional to cell concentration; additionally, mutual shading can cause light insufficiency which leads to low lipid content and algal biomass [12,13]. Light limitation can be overcome by the cultivation of microalgae under heterotrophic conditions, meaning supplementation of growth media with organic carbon to meet cellular energy requirements [14]. The appeal of heteroptrophic microalgae cultivation is the elimination of light requirements which can be expensive and limit growth of cell cultures [15]. Heterotrophic growth of algae cultures can result in higher production of biomass and accumulation of high lipid content in cells [16]. The highest biomass achieved in a photoautotrophic system has been reported in *Chlorella* sp. (40 g/L dry cell weight, DCW) using thin layer cultures [17]. In heterotrophic fedbatch cultures *Chlorella* concentrations ranging from 100 to ≥150 g/L are achievable [15]. However, only a discrete number of algae have the native capacity to utilise external carbon sources for growth, limiting the utility of this culture method.

The combination of heterotrophic capability plus the ability to produce high value products in significant quantities is essential for large-scale commercial exploitation of microalgae. A unicellular diatom *P. tricornutum* naturally accumulates high levels of EPA and has emerged recently as a potential source for its industrial production. It has a rapid growth rate and can accumulate lipids in the form of triacylglycerols (TAGs) up to 30% of its dry cell weight. However, native strains accumulate only traces of DHA.

The engineering of the photoautotrophic diatom *P. tricornutum* to produce a heterotrophic strain has been described previously [18]. A trophic conversion was achieved by transformation of this alga with a single gene encoding a glucose transporter (*glut1*). This study demonstrated the potential for commercial cultivation of *P. tricornutum* under heterotrophic conditions, and also emphasised that, in the absence of appropriate carbon transporters and metabolic pathways, exogenous sugars cannot be utilised by some strains for growth.

Recently we have engineered the *P. tricornutum* strain, Pt_Elo5, to accumulate elevated levels of DHA, making it particularly promising for the biotechnological production of omega-3 LC-PUFAs [19]. Transgenic cells overexpressing a C20 Δ5-elongase from the picoalga *Ostreococcus tauri,* OtElo5 [20], demonstrated an eightfold increase in DHA production compared to wild type (WT). Importantly, EPA and DHA accumulated in the form of TAGs, a key requirement for the extraction of these high value lipids from the cells.

In this study, we have used a multigene vector to further improve the commercial potential of *P. tricornutum* by engineering a transgenic strain that accumulates high levels of DHA and is able to grow heterotrophically. *P. tricrornutum* cells have been transformed with a multigene cassette containing two different genes: OtElo5 encoding a Δ5-elongase from *O. tauri*, [20] and a putative glucose transporter gene from *Physcomitrella patens*, (*Ppglut1*)*.* The effects of glucose concentrations on growth and LC-PUFA production of wild type and transformed strains cultivated under photoautotrophic, heterotrophic and mixotrophic growth conditions were studied. We demonstrate that the transgenic cells grown in the light accumulate higher biomass when supplemented with glucose and contain elevated levels of EPA and DHA. This study is the first report of a metabolic engineering of heterotrophic microalga to produce enhanced levels of both EPA and DHA.

## 2. Results and Discussion

### 2.1. Trophic Conversion of Transgenic Pt_Elo5 Strain Accumulating Enhanced Levels of DHA

A multigene vector containing the OtElo5 gene and a glucose transporter*,* Ppglut was used to transform *P. tricornutum* via biolistics. Following selection on zeocin containing plates, twenty four clones were sub-cultured and used for analysis of fatty acids by gas chromatography coupled with a flame ionization detector (GC-FID). The analyses of fatty acid methyl esters (FAMEs) revealed that nine clones contained docosapentaenoic acid (DPA, 22:5*n*-3), a product of EPA elongation by a C20 Δ5-elongase from *O. taurii* and the direct precursor of DHA. The presence of both genes in these clones was confirmed by PCR amplification of the OtElo5 and Ppglut1 from genomic DNA. Two independent transgenic strains that grew successfully in the dark in Enriched ESAW (EE) media supplemented with 1% glucose were selected for further analyses.

The two heterotrophic clones, Pt_HElo5_5 and Pt_HElo5_7 were grown in the light and dark in enriched ESAW medium supplemented with glucose and their fatty acid profiles analysed (Table 1).

Transgenic strain Pt_Elo5, over-expressing the transgene OtElo5 was used as the control in this study. In the light, Pt_Elo5 cells grown in the presence of 1% glucose accumulated elevated levels of DHA (6.3%) compared to that of the wild type, WT (1.8%). WT and Pt_Elo5 cells were unable to grow in the dark. Light grown cultures of Pt_HElo5_5 and Pt_HElo5_7 accumulate elevated levels of DHA (7.5% and 8.1% respectively) compared to Pt_Elo5.

Under heterotrophic conditions, Pt_HElo5_5 and Pt_HElo5_7 strains both accumulated slightly reduced DHA levels compared to light grown (7.3% and 5.2%, respectively). This may indicate that accumulation of DHA in *P. tricornutum* requires light. Interestingly, dark grown cultures of Pt_HElo5_5 and Pt_HElo5_7 accumulated higher levels of DPA (5.2% and 9.1%, respectively) compared to cultures grown in the light (3.3% and 1.8% DPA, respectively). This may be due to the enhanced activity of OtElo5 in the dark and demonstrates the efficiency of the *fcp* promoter in the dark. Dark conditions did not reduce EPA levels in either strain. Moderate decrease of DHA content may be the result of reduced activity of Δ4-desaturase in the dark.

The final Δ5-elongation and Δ4-desaturation steps required for *n*-3 C20/C22 LC-PUFA formation have been identified as light dependent in the marine microalga *Pavlova lutheri* [21]*.* The highest elongation and desaturation conversion rates were measured under low light. Chauton, *et al.* reported that several genes encoding desaturases involved in the biosynthesis of unsaturated PUFAs in *P. tricornutum* reached maximum expression levels after the onset of light and were the most responsive to the dark/light transition [22].

Dark grown cultures of Pt_HElo5_5 and Pt_HElo5_7 also demonstrate a build-up of 18:1 (11.7% and 7.7% compared to 2.8% and 1.8% in the light, respectively) and linoleic acid (LA; 18:2*n*-6) (6.3% and 1.4% compared to 1.0% and 0.7% in the light, respectively). This could be associated with increased activities of endogenous Δ9- and Δ12-desaturases.

In agreement with previous observations (for Refs, see [23]), under heterotrophic conditions the production of C16:0 is increased in the both transgenic clones while C16:1 content is markedly reduced.

Pt_HElo5_5 cells accumulated slightly higher DHA than Pt_HElo5_7 cells (7.3% compared to 5.2%) under heterotrophic conditions and so were taken further for more detailed analysis.

### 2.2. Impact of a Glucose on Cell Growth

**Figure 1:**
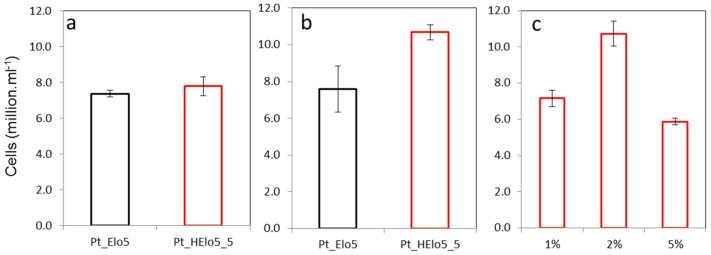
Cellular growth of transgenic cells. (**a**) in the light (*p* = 0.491, SED = 0.551 on 4df, *t*-test); (**b**) in the light plus glucose (*p* = 0.080, SED = 1.319 on 4df, *t*-test); (**c**) in the dark plus glucose (*p* < 0.001, SED = 0.683 on 6df, LSD (5%) = 1.672, ANOVA: 1% and 5% glucose significantly different from 2%, and *p* < 0.05, LSD. No significant differences between 1% and 5%). Values are the average of three biological replicates (±standard error).

Pt_Elo5 and Pt_HElo5_5 cells were grown in EE media in the presence of glucose and cell number was recorded in the stationary (S) phase of growth and compared to that in the cultures grown without glucose (Figure 1). In the absence of glucose, Pt_HElo5_5 cells grew to around the same cell density as Pt_Elo5 cells. The addition of glucose to the growth media resulted in higher cell number in Pt_HElo5_5 compared to Pt_Elo5 cells (10.7 and 7.8 million cells/mL, respectively). Pt_HElo5_5 cells grown in the dark and supplemented with 1% glucose grew to the same cell density as photoautotrophic cultures without glucose. Increasing glucose concentration to 2% *w*/*v* boosted cell number to the equivalent of mixotrophic cultures. A further increase in glucose to 5% reduced cell number to around 5.9 million cells/mL*.*

### 2.3. Analyses of Fatty Acid Composition of Pt_HElo5_5 Cells Grown at Different Glucose Concentrations

**Figure 2:**
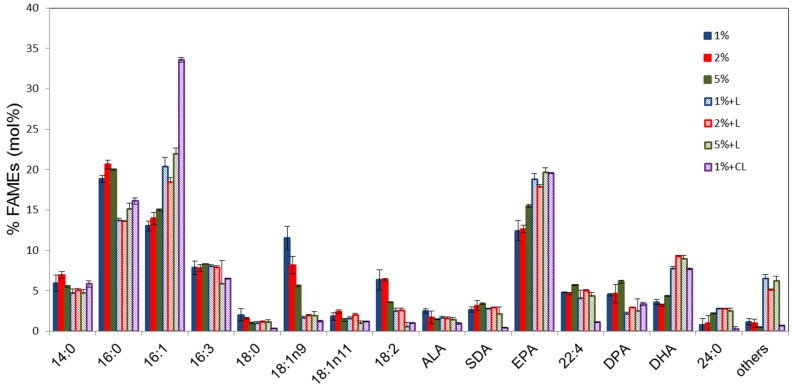
Fatty acid composition of transgenic Pt_HElo5_5 cells grown under different glucose concentrations in the dark and in the light. % indicates glucose concentration; +L, dark grown cells transferred to light conditions for 24 h; CL, cells grown under constant light. Values are the average of three biological replicates (±standard error). For quantitative statistical analysis, see Supplementary Table S1.

Total fatty acid methylesters (FAMES) of Pt_HElo5_5 cells grown under heterotrophic conditions at different glucose concentrations were analysed by GC-FID (Figure 2). The fatty acid (FA) profiles of the cells at different glucose concentrations are similar although C18:1 appears to accumulate in higher levels in cells grown in 1% glucose (up to 13.4%) and decrease with increasing glucose concentration (average 7.2% in the presence of 5% glucose). The levels of LC-PUFAs in cells grown in 5% glucose are slightly higher compared to those accumulated in the presence of 1% and 2% glucose. Interestingly, unlike under photo- and mixo-trophic conditions, C22:4*n*-6 and DPA levels were higher at each glucose concentration than DHA levels.

Following heterotrophic growth to the S phase, cultures were transferred to the light for 24 h and total FA measured. The period of light incubation substantially altered the FAME profiles at all concentrations of glucose. DHA levels in dark grown cultures transferred to the light were higher than Pt_Elo5, between 7.8% and 9.2%. EPA levels are also higher than in Pt_Elo5 cells. There was a significant decrease in 16:0 correlated with an equivalent increase in 16:1. The levels of C18 fatty acids α-linolenic (ALA; 18:3*n*-3) and stearidonic (SDA; 18:4*n*-3) remained largely unchanged whilst levels of 18:1*n*-9 and 18:2*n*-6 reduced considerably following light treatment. The reduction in medium chain PUFAs correlates with an increase in EPA and DHA. Levels of 22:4*n*-6 remained unchanged following light treatment but DPA levels were reduced.

Mixotrophic growth of *P. tricornutum* has been previously reported. A fed batch approach was used to promote high biomass compared to photoautotrophic growth, however, under these conditions, EPA accumulation was lower than photoautotrophic [24]. In contrast, Pt_HElo5_5 cells grown mixotrophically accumulated more EPA and DHA than photo- or heterotrophically.

### 2.4. Quantification of LC-PUFAs Accumulation in Cells under Photo-, Mixo- and Heterotrophic Conditions

An internal standard was used to quantify the amount of EPA, DPA and DHA accumulating in the S phase under each growth condition of the control Pt_Elo5 and Pt_HElo5_5 cells (Table 2). Under photoautotrophic conditions, Pt_HElo5_5 cells accumulated the same amount of EPA as the control cells, and slightly lower amounts of DHA (2.48 μg/mg dry weight compared to 3.6 μg/mg). Supplementation of the growth media with 1% glucose resulted in slightly lower accumulation of EPA and DHA in Pt_Elo5 cells and an increase in EPA and DHA in Pt_HElo5_5 cells. EPA levels in Pt_HElo5_5 cells were 1.7 fold higher than those in Pt_Elo5 cells. DHA content of Pt_HElo5_5 cells was 1.1 fold higher compared to that in Pt_Elo5.

Under heterotrophic conditions, levels of EPA and DHA were reduced compared to mixo- and photoautotrophic growth. This reduction is consistent at each glucose concentration—so is not glucose dependent. EPA levels were around 50% lower than under mixotrophic conditions and DHA levels around 60% lower.

### 2.5. Comparison of Lipid-Bound FAs in Cells Grown under Photo, Mixo and Heterotrophic Conditions

Solid phase extraction was used to separate total lipid extracts. The accumulation of EPA, DPA and DHA was then determined using GC-FID. In accordance with previously reported observations [25], Pt_Elo5 cells accumulate EPA mainly in the glycolipid fraction, GL, (56%) (Table 3). The remaining EPA was distributed between neutral lipids, NL, (23.8%) and phospholipids, PL (17.3%). Addition of glucose to the media did not have a significant impact on this pattern of lipid distribution in Pt_Elo5 cells.

Pt_HElo5_5 cells grown under photoautotrophic conditions accumulated a higher proportion of EPA in neutral lipids (36.5%) compared to that in Pt_Elo5 cells (23.8%). The amount of EPA in the phospholipid fraction of Pt_HElo5_5 cells (15.4%) was similar to that of Pt_Elo5 (17.3%), whilst a lower level was observed in the glycolipid fraction (48.1% compared to 58.9%). There was also a higher proportion of DHA in the NL than in Pt_Elo5, 23.6% compared to 12.9%. A higher proportion of DHA was also observed in the GL, 13.9% compared to 8.5% in Pt_Elo5. DHA had a lower distribution in the PL (62.5%) compared to Pt_Elo5 (78.6%).

Addition of 1% glucose to the media had an impact on lipid distribution in Pt_HElo5_5 cells. EPA levels were reduced in the NL compared to photoautotrophic conditions, 18.7% compared to 36.5%. The highest proportion of EPA was found in the GL fraction with 25% in the PL. DHA distribution looked more similar to Pt_Elo5 as DHA is principally found in the PL (80.4%) followed by NL (12.5%) with the lowest accumulation in GL (7.1%). Interestingly the FA distribution was very similar between mixo- and heterotrophic conditions in Pt_HElo5_5 cells.

Further engineering of the Pt_HElo5_5 strain is required to boost the DHA content in TAG. Candidate genes may include diacylglycerol acyltransferase (DGAT) genes involved in TAG biosynthesis.

## 3. Experimental Section

### 3.1. Strains and Growth Conditions

*P. tricornutum* UTEX646 (Pt4) was grown in ESAW medium supplemented with enriched phosphate and nitrate (EE) according to Zaslavaskaia, *et al.*, 2000 [18]. (Sterile filtered glucose was added to cultures post-autoclaving. Cells were grown at 20 °C and agitated at 60 rpm under white fluorescent lights’ constant illumination (60 μmol photons m^−2^·s^−1^). Dark conditions were achieved by growing cultures in a black box. Cells grown mixotrophically were grown under constant illumination as in photoautotrophic conditions, but media was supplemented with 1% glucose.

### 3.2. Plasmid Design and Transformation

A double vector was constructed by modifying the pPhaT-1 vector as described in Hamilton, *et al.*, 2014 [19]. An OtElo5 gene was inserted into the vector following digestion with *Kpn*I*,* and *Sac*I. Glut1 from *Physcomitrella patens* was introduced following digestion with *Bam*HI and *Xba*I. Plasmids were transformed into *P. tricornutum* as described previously [19]. Positive transformants were identified by antibiotic selection and confirmed by PCR. These clones were then streaked on to EE plates containing 1% glucose and transferred to the dark to test for heterotrophic growth.

### 3.3. Biomass Determination

Dry weight of cells was measured by harvesting cells by centrifugation, washing with 3% ammonium formate to remove ASH, snap freezing and freeze drying. Cells were then weighed.

### 3.4. Fatty Acid Analysis

Algal cells were harvested by centrifugation at 3500 *g* for 15 min. Fatty acids were extracted and methylated as described [26] with minor modifications. A 15 mL aliquot of algal culture was harvested; following methylation the heptane fraction was concentrated and re-suspended in 40 μL solvent prior to injection of 1 μL on to the GC column. Methyl ester derivatives of total fatty acids extracted were analysed by GC (Agilent 7890A, Santa Clara, CA, USA) using an Agilent DB-225 column and identified using known 37 FAMES mix standards (Sigma-Aldricht, Haverhill, UK).

### 3.5. Lipid Analysis

Total lipid was extracted using a modified Bligh and Dyer method [27]. Freeze dried cells (10 mg) from the stationary growth phase were heated in 1 mL propan-2-ol at 80 °C for 10 min. The solvent fraction was retained and the cell pellet homogenised in a total volume of 6 mL chloroform:methanol (2:1). Following centrifugation the cell pellet was re-extracted with 4 mL chloroform:methanol (2:1). Lipid extract was dried under nitrogen and re-suspended in 0.5 mL chloroform. Lipid extract was fractionated into neutral lipids, glycolipids and phospholipids using Sep-pak columns as described previously [28]. Fatty acids were methylated and analysed by GC-FID. 17:0 fatty acid was used as an internal standard.

### 3.6. Quantitative Analysis

Student’s *t*-tests and Analysis of Variance (ANOVA) was applied to the data on cellular growth. Where appropriate, a control plus factorial structured ANOVA was applied to the data on fatty acid composition and categorisations of EPA, DPA and DHA, or otherwise one-way ANOVA was used. Standard error of the difference (SED) values were provided, based on the residual degrees of freedom (df) from the ANOVA, for comparison of means using the corresponding least significant difference (LSD) values at the 5% (*p* < 0.05) level of significance. The GenStat statistical package (17th edition, ©VSN International Ltd., Hemel Hempstead, UK) was used for these analyses.

## 4. Conclusions

Photoautotrophic growth for the commercial production of high value products from algae is limited by the costs associated with culture conditions and biomass [29,30]. Growth of heterotrophic strains, which are either wild type (WT) or engineered, allows the circumvention of this bottleneck. Marine diatom *P. tricornutum* is an excellent candidate for high value lipid production due to its rapid growth cycle, sequenced genome and high lipid content. However, its inability to grow under heterotrophic conditions limits its potential as a commercial strain.

This study is the first report of a heterotrophic microalga producing enhanced levels of both EPA and DHA. We have performed a trophic conversion of transgenic *P. tricornutum* strain accumulating both EPA and DHA by transforming it with a OtElo5 gene encoding a Δ5-elongase from *O. tauri* and *Ppglut1*, encoding a moss glucose transporter. This metabolically engineered strain has the capacity to grow on glucose in the dark and accumulate substantial levels of omega-3 LC-PUFAs. The highest omega-3 LC-PUFAs accumulation was observed in cultures grown under mixotrophic conditions in the presence of 1% glucose (up to 32.2% of total fatty acids, TFA). Both DHA and EPA are detected at high levels in the neutral lipids of transgenic cells grown under phototrophic conditions, averaging 36.5% and 23.6% of TFA, respectively. Such heterotrophic growth has the potential to improve the efficiency and reduce the cost of microalgal biomass production.

## Figures and Tables

**Table 1:** Fatty acids composition of wild type (WT) and transgenic Pt_Elo5, Pt_HElo5_5 and Pt_HElo5_7 cells grown in the presence of 1% glucose in the light (+L) or in the dark (−L). Values are the average of three biological replicates (±standard error).

	14:0	16:0	16:1	16:3	18:0	18:1	18:2	EPA	DPA	DHA	Others
WT + L	5.2 ± 0.1	14.6 ± 0.2	38.5 ± 1.4	2.7 ± 0.1	0.4 ± 0.1	7.4 ± 1.1	0.3 ± 0.2	24.7 ± 0.6	nd	1.8 ± 0.1	2.7 ± 0.1
Pt_Elo5 + L	5.1 ± 0.2	14.1 ± 0.5	35.1 ± 1.2	7.7 ± 0.3	1.5 ± 0.1	2.5 ± 0.1	0.7 ± 0.1	15.9 ± 0.5	1.9 ± 0.3	6.3 ± 0.1	11.6 ± 1.7
Pt_HElo5_5 + L	5.7 ± 1.0	15.6 ± 0.4	32.6 ± 0.6	6.3 ± 0.8	0.3 ± 0.1	2.8 ± 0.5	1.0 ± 0.1	19.0 ± 1.2	3.3 ± 0.2	7.5 ± 0.3	5.1 ± 0.4
Pt_HElo5_5 − L	7.1 ± 0.9	23.4 ± 1.3	22.4 ± 3.8	8.2 ± 1.8	2.0 ± 0.7	11.7 ± 2.9	6.3 ± 1.8	19.9 ± 2.6	5.2 ± 0.6	7.3 ± 1.8	14.2 ± 2.3
Pt_HElo5_7 + L	6.1 ± 0.5	11.8 ± 0.7	34.5 ± 3.1	7.0 ± 0.5	1.4 ± 0.2	1.8 ± 0.3	0.7 ± 0.1	11.9 ± 0.7	1.8 ± 1.1	8.1 ± 0.7	7.9 ± 0.4
Pt_HElo5_7 − L	9.5 ± 0.6	22.1 ± 0.2	13.8 ± 0.1	7.1 ± 0.1	1.0 ± 0.1	7.7 ± 0.1	1.4 ± 0.1	13.9 ± 0.1	9.1 ± 2.3	5.2 ± 0.3	5.7 ± 0.2
F-test (*p*-values)	0.217	0.002	0.045	<0.001	<0.001	<0.001	0.159	<0.001	<0.001	<0.001	<0.001
SED	- *	0.70	2.58	0.40	0.20	0.79	-	0.72	0.18	0.81	0.19
df	-	8	8	8	8	8	-	8	6	8	8
LSD (5%)	-	1.60	5.96	0.91	0.40	1.83	-	1.65	0.44	1.87	0.43

* No significant (*p* < 0.05, *F*-test) difference between treatments, so no SED required for comparisons of means.

**Table 2:** Accumulation of EPA, DPA and DHA in transgenic cells grown in the light (L); in the light plus glucose (L + G); in the dark plus glucose (−L + G). Values are the average of three biological replicates (±standard error).

Cells	LC-PUFA μg/mg Dry Weight		
	EPA	DPA	DHA
Pt_Elo5/L	6.0 ± 0.8	1.5 ± 0.1	3.6 ± 0.8
Pt_HElo5_5/L	6.0 ± 1.0	1.1 ± 0.3	2.5 ± 0.3
Pt_Elo5/L + 1% G	4.7 ± 0.2	1.1 ± 0.1	2.8 ± 0.2
Pt_HElo5_5/L + 1% G	8.0 ± 0.5	1.4 ± 0.1	3.1 ± 0.2
Pt_HElo5_5/−L + 1% G	3.4 ± 0.2	1.3 ± 0.3	1.0 ± 0.1
Pt_HElo5_5/−L + 2% G	3.6 ± 0.1	1.7 ± 0.1	1.0 ± 0.1
Pt_HElo5_5/−L + 5% G	3.3 ± 0.4	1.3 ± 0.2	1.0 ± 0.1
*F*-test (*p*-values)	<0.001	0.302	<0.001
SED	0.76	- *	0.47
df	14	-	14
LSD (5%)	1.63	-	1.00

* No significant (*p* < 0.05, *F*-test) difference between treatments, so no SED required for comparisons of means.

**Table 3:** Lipid analyses of transgenic cells grown in the light (L); in the light plus glucose (L + G); in the dark plus 1% glucose (−L + G). Values are the average of three biological replicates (±standard error). For quantitative statistical analysis, see Supplementary Table S2.

Strain	NL			GL			PL		
	EPA	DPA	DHA	EPA	DPA	DHA	EPA	DPA	DHA
Pt_Elo5/L	23.8 ± 7.4	16.2 ± 7.7	12.9 ± 3.0	58.9 ± 6.4	7.7 ± 4.2	8.5 ± 2.5	17.3 ± 4.0	76.1 ± 12.3	78.6 ± 17.1
Pt_HElo5_5/ L	36.5 ± 4.8	32.9 ± 4.6	23.6 ± 1.7	48.1 ± 9.6	12.8 ± 2.3	13.9 ± 1.3	15.4 ± 4.5	54.3 ± 24.6	62.5 ± 6.9
Pt_Elo5/ L + G	20.2 ± 2.3	14.5 ± 2.3	11.9 ± 2.3	63.9 ± 12.0	5.9 ± 0.9	6.7 ± 1.4	15.9 ± 1.6	79.5 ± 8.3	81.4 ± 13.5
Pt_HElo5_5/L + G	18.7 ± 0.8	17.3 ± 0.7	12.5 ± 0.1	56.1 ± 2.1	6.5 ± 0.2	7.1 ± 0.2	25.2 ± 1.0	76.1 ± 4.0	80.4 ± 2.3
Pt_HElo5_5/−L + G	19.1 ± 2.5	16.1 ± 2.2	10.9 ± 0.8	56.8 ± 0.1	11.8 ± 1.6	14.9 ± 3.8	24.1 ± 5.9	72.2 ± 5.9	74.1 ± 16.6

## Supplementary Files

Supplementary File 1
