# Supplementary material for: Heterotrophic Production of Omega-3 Long-Chain Polyunsaturated Fatty Acids by Trophically Converted Marine Diatom Phaeodactylum tricornutum
Source: Mar Drugs. 2016 Mar 9;14(3):53. doi: 10.3390/md14030053 (PMC4820307; doi:10.3390/md14030053)
Supplement: Supplementary File 1 [file marinedrugs-14-00053-s001.pdf]

Supplementary Materials: Heterotrophic Production of Omega-3 Long-Chain Polyunsaturated Fatty Acids by Trophically Converted Marine Diatom *Phaeodactylum tricornutum*

Mary L. Hamilton, Stephen Powers, Johnathan A. Napier and Olga Sayanova

Table S1. Quantitative statistical analysis of fatty acid composition of transgenic Pt_HElo5_5 cells grown under different glucose concentrations in the dark and in the light (see Figure 2). There is a control + factorial structure for this experiment, so we present *p*-values for the main effects and interactions between light and glucose.

F-test Results (*p*-Values)	14:0	16:0	16:1	16:3	18:0	18:1n9	18:1n11	18:2	ALA	SDA	EPA	22:4	DPA	DHA	24:0	others
Main effect of Control *vs.* treated	0.514	0.075	<0.001	0.373	0.014	<0.001	0.084	<0.001	0.063	<0.001	<0.001	<0.001	0.59	<0.001	0.006	<0.001
Main effect of Glucose	0.245	0.03	0.014	0.727	0.392	0.005	0.005	<0.001	0.249	0.713	0.005	0.408	0.41	0.004	0.497	0.176
Main effect of Light	0.006	<0.001	<0.001	0.435	0.086	<0.001	0.081	<0.001	0.251	0.182	<0.001	0.122	<0.001	<0.001	0.005	<0.001
Interaction of Glucose and light	0.582	0.05	0.122	0.457	0.265	0.003	0.93	0.737	0.444	0.292	0.261	0.17	0.438	0.005	0.22	0.155

$p < 0.05$ ANOVA terms to interpret in yellow (terms with $p < 0.05$ above these are superseded by these in yellow).

Table S2. Quantitative statistical analysis of transgenic cells grown in the light (L); in the light plus glucose (L + G); in the dark plus 1% glucose (−L + G) (Table 3). There is a control + factorial structure for this experiment (Table 3), so we present *p*-values for the main effects and interactions between strains and light.

Lipid Class	NL			GL			PL		
Fatty Acid	EPA	DPA	DHA	EPA	DPA	DHA	EPA	DPA	DHA
F-test Results (*p*-values)	NL_EPA	NL_DPA	NL_DHA	GL_EPA	GL_DPA	NL_DHA	PL_EPA	PL_DPA	PL_DHA
Main effect of No light control	0.259	0.402	0.070	0.994	0.198	0.037	0.224	0.966	0.914
Main effect of Line	0.220	0.046	0.014	0.244	0.244	0.215	0.356	0.365	0.515
Main effect of Glucose	0.030	0.072	0.009	0.406	0.110	0.079	0.304	0.362	0.431
Interaction of Line with Glucose	0.126	0.136	0.023	0.845	0.357	0.279	0.18	0.505	0.562

$p < 0.05$ ANOVA terms to interpret in yellow (terms with $p < 0.05$ above these are superseded by these in yellow).
